# 4127 Achieving health equity in translational research: Applying critical race theory in workforce curricula to address disparity

**DOI:** 10.1017/cts.2020.250

**Published:** 2020-07-29

**Authors:** Kristina Gern Johnson, Karen C. Johnston, Jennifer Phillips, Maryellen Gusic

**Affiliations:** 1 University of Virginia; 2 iTHRIV

## Abstract

OBJECTIVES/GOALS: Learners will:

- Identify social structures that serve as root causes of health disparities
- Critically evaluate the ways in which racism, culture, and power perpetuate disparity
- Use critical reflection to shape their research and advocate for institutional change

METHODS/STUDY POPULATION: The Integrated Translational Health Research Institute of Virginia (iTHRIV) Health Equity curriculum provides a lens for participants to view health disparities, social structures that create and perpetuate disparities, and the path to a more equitable future. This longitudinal workforce curriculum incorporates the principles of critical race theory (CRT), including: race as a social construct, structural determinism, intersectionality, and the social construction of knowledge. Learners gain practical experience through facilitated group discussions and critical reflection of their own work including research question design, recruitment, dissemination, and enhancing the faculty pipeline. RESULTS/ANTICIPATED RESULTS: To measure the impact of the curriculum, we will evaluate learners’ participation in mentoring activities for persons from underrepresented backgrounds; participation in local and national diversity and inclusion efforts; engagement in community-based research; ability to account for implicit bias and power imbalances in their research design, including in recruitment and retention; and share research findings with community members and research participants. Evaluation strategies will include quantitative and qualitative methodologies. DISCUSSION/SIGNIFICANCE OF IMPACT: There is growing recognition of the impact of racism on the development and perpetuation of health disparities. Public health critical race praxis (an adaptation of CRT) is emerging as a theoretical framework to empower researchers to challenge the status quo in order to achieve health equity.

